# Accelerated Brain Aging in Patients With Obsessive-Compulsive Disorder

**DOI:** 10.3389/fpsyt.2022.852479

**Published:** 2022-05-06

**Authors:** Liang Liu, Junhong Liu, Li Yang, Baohong Wen, Xiaopan Zhang, Junying Cheng, Shaoqiang Han, Yong Zhang, Jingliang Cheng

**Affiliations:** ^1^Department of Magnetic Resonance Imaging, The First Affiliated Hospital of Zhengzhou University, Zhengzhou, China; ^2^Department of Public Health, School of Medicine, Huanghuai University, Zhumadian, China

**Keywords:** obsessive-compulsive disorder, brain age, structural brain imaging, machine learning, gray matter volume (GMV)

## Abstract

Obsessive-compulsive disorder (OCD) may be accompanied by an accelerated structural decline of the brain with age compared to healthy controls (HCs); however, this has yet to be proven. To answer this question, we built a brain age prediction model using mean gray matter volumes of each brain region as features, which were obtained by voxel-based morphometry derived from T1-weighted MRI scans. The prediction model was built using two Chinese Han datasets (dataset 1, *N* = 106 for HCs and *N* = 90 for patients with OCD; dataset 2, *N* = 270 for HCs) to evaluate its performance. Then, a new prediction model was trained using data for HCs in dataset 1 and applied to patients with OCD to investigate the brain aging trajectory. The brain-predicted age difference (brain-PAD) scores, defined as the difference between predicted brain age and chronological age, were calculated for all participants and compared between patients with matched HCs in dataset 1. It was demonstrated that the prediction model performs consistently across different datasets. Patients with OCD presented higher brain-PAD scores than matched HCs, suggesting that patients with OCD presented accelerated brain aging. In addition, brain-PAD scores were negatively correlated with the duration of illness, suggesting that brain-PAD scores might capture progressive structural brain changes. These results identified accelerated brain aging in patients with OCD for the first time and deepened our understanding of the pathogenesis of OCD.

## Introduction

Obsessive-compulsive disorder (OCD), a debilitating psychiatric disorder with an estimated lifetime prevalence of 1–3% (1) is characterized by uncontrollable obsessions and compulsions (2). OCD affects approximately 3% of the total population worldwide, and its symptoms impose a great burden on society (3). Despite significant advances in the study of OCD, the underlying pathogenesis remains unknown.

In clinical, OCD is highly comorbid with psychiatric diseases such as schizophrenia and bipolar disorder (4). For example, obsessive–compulsive symptoms are frequently observed in patients with schizophrenia (5). Up to 30% of patients with schizophrenia show obsessive-compulsive symptoms, and 12% of patients with schizophrenia satisfy the obsessive-compulsive diagnostic criteria for OCD (6, 7). Comorbidity is also found between OCD and depression, as well as bipolar disorder (8–10). These studies suggest that the pathogenesis of OCD overlaps with these diseases to some extent. Mental disorders are accompanied by an accelerated aging trajectory, supported by abundant evidence from functional and biological states (11–17). New evidence from recent studies combining neuroimaging and machine learning methods to calculate a so-called brain age while handling the variation in brain structures between individuals confirms this notion (18–20). Moreover, exploring abnormal brain development trajectories by means of brain age helps identify clinically indistinguishable diseases. For example, the brain age method outperforms other state-of-the-art biomarkers with an accuracy rate of 81% when identifying mild cognitive impairment (21), and the method aids early differential diagnosis between schizophrenia and bipolar disorder (18). The accelerated brain aging is also found in other brain disorders such as depression (22), epilepsy (23) autism (24) and Alzheimer's disease (25). In fact, accelerated white matter decline with age is observed in patients with OCD, suggesting that brain aging might also be accelerated in patients with OCD (26). However, whether brain aging is accelerated in OCD remains unclear.

In the current study, we explored whether the brain development trajectory was abnormal in OCD using brain gray matter volumes combined with a machine learning method for the first time. We first built a brain age prediction model where mean gray matter volumes of the brain region were treated as features using Gaussian process regression (GPR) (27). The model was trained using healthy subjects in two independent datasets (*N* = 130 for healthy controls (HCs) in dataset 1; *N* = 270 in dataset 2) and then applied to infer the brain ages of patients with OCD. The brain-predicted age difference (brain-PAD) scores, defined as the difference between predicted brain age and chronological age, were calculated in patients with OCD and then compared with age- and sex-matched HCs in dataset 1. A positive brain-PAD score reflects accelerated predicted brain aging, while a negative score represents that a predicted brain age younger than the chronological age norm. There were two assumptions in this study. First, considering the accelerated white matter decline with age observed in OCD, which suggests that brain aging is accelerated in patients with OCD (26), we expected to find higher brain-PAD scores in patients with OCD than in matched HCs. Second, we hypothesized that the brain-PAD scores in OCD were lower than those reported in schizophrenia (18, 20), due to the greater neurological abnormalities in schizophrenia compared with OCD (28–30).

## Materials and Methods

### Sample

#### Dataset 1

Patients with OCD were recruited from the outpatient services of the Department of Psychiatry of the First Affiliated Hospital of Zhengzhou University, Zhengzhou, China. Patients were diagnosed according to the Diagnostic and Statistical Manual of Mental Disorders, Fifth Edition (DSM-V) for OCD, which was performed by two psychiatrists. The inclusion criteria were as follows: (1) first episode; (2) never having taken any antidepressants, antipsychotics, or other treatment; (3) Han Chinese ethnicity and right-handedness; (4) 18 to 50 years of age; and (5) primary school education or above. The exclusion criteria were as follows: (1) comorbidity with other mental disorders; (2) history of nervous system diseases such as brain tumor, epilepsy, or brain trauma; and (3) first-degree relatives suffering from mental illness or neurological disease. The Yale–Brown Obsessive Compulsive Scale (Y-BOCS) was used to evaluate symptom severity (31).

HCs were recruited from the community through poster advertisements. All HCs were Han Chinese and were right-handed.

The exclusion criteria for all participants (both HCs and OCD patients) were as follows: (1) use of drugs, such as anesthesia, sleeping, and analgesia, in the past month; (2) history of substance abuse; (3) history of brain tumor, trauma, surgery, or other organic body diseases; (4) history of cardiovascular disease, diabetes, or hypertension; (5) contraindications to MRI scanning, including fixed dentures, metal braces, artificial heart valves, and other metal foreign bodies in the body; and (6) other structural brain abnormalities revealed by MRI scans.

Written informed consent was obtained from all participants prior to the experiment. The study was approved by the research ethics committee of the First Affiliated Hospital of Zhengzhou University.

#### Dataset 2

The second dataset comes from the Southwest University Adult Lifespan Dataset available for research purposes [(http://fcon_1000.projects.nitrc.org/indi/retro/sald.html). Dataset 2 included 492 healthy participants [306 female, 186 male; age range 19–90 years]. The exclusion criteria included MRI-related exclusion criteria, current psychiatric or neurological disorders, use of psychiatric drugs in the past 3 months prior to scanning, in addition to other criteria. A detailed description of the participant information and data acquisition parameters is provided in a previous study (32). Due to the age range for dataset 1 of 18–50 years, the current study only included participants under the age of 50 years (*N* = 270) in dataset 2.

### Data Acquisition

#### Dataset 1

T1-weighted anatomical images of participants in dataset 1 were acquired using on 3-Tsela GE Discovery MR750 scanner (General Electric, Fairfield Connecticut, USA). Using an 8-channel prototype quadrature birdcage head coil, we acquired structural T1-weighted images with 3D spoiled gradient echo scan sequence: Repetition time = 8,164 ms, echo time = 3.18 ms, inversion time = 900 ms, flip angle = 7 degrees, resolution matrix = 256 × 256, slices = 188, thickness = 1.0 mm, and voxel size = 1 × 1 × 1 mm^3^).

#### Dataset 2

High-resolution T1-weighted anatomical images of participants in dataset 2 were acquired using a magnetization-prepared rapid gradient echo (MPRAGE) sequence (repetition time = 1,900 ms, echo time = 2.52 ms, inversion time = 900 ms, flip angle = 90 degrees, resolution matrix = 256 × 256, slices = 176, thickness = 1.0 mm, and voxel size = 1 × 1 × 1 mm^3^) (32).

### Voxel-Based Morphometry Analysis

To obtain the structural volumes of gray matter, voxel-based morphometry was adopted following the standard pipeline of the CAT12 toolbox (http://dbm.neuro.uni-jena.de/cat12/). The main steps included bias-field correction, segmentation (gray and white matter and cerebrospinal fluid), adjustment for partial volume effects, normalization into the Montreal Neurological Institute space, resampling to 1.5 mm × 1.5 mm × 1.5 mm, and non-linear modulation (33). Finally, the gray matter maps were smoothed with a 6 mm full width at half maximum Gaussian kernel. The total intracranial volume (TIV) of each participant was also calculated for comparative analysis, and its effect on brain-PAD score was investigated.

### Brain Age Prediction Model

As done in previous study (27), GPR was employed to infer the brain age of each participant, where the mean gray matter volumes of brain regions were defined in 246 regions of interest of the Brainnetome atlas (34). The GPR used in the current study was implemented in the Gaussian processes for machine learning (GPML) toolbox (www.gaussianprocess.org/gpml/code/). The model parameters were optimized using a conjugate gradient optimizer (also included in the GPML toolbox), as previously described (35, 36). To exclude the influence of atlas selection on our results, an atlas with 200 regions of interest was chosen to validate the performance of the prediction model (37).

### Model Validation

Ten-fold cross-validation (CV) was used to evaluate the performance of the prediction model (23, 38). To evaluate the performance of the prediction model, we calculated the (1) mean absolute error (MAE) between the estimated brain age and chronological age, and (2) the correlation between the chronological age and estimated brain age across 10-fold CV. To eliminate the effect of randomization on our results, this procedure was repeated 100 times. In this procedure, two datasets were used to evaluate the performance of the prediction model.

### Statistics

After confirming the performance of the brain age prediction model, a new prediction model was built with HCs in dataset 1 and applied to patients with OCD. The brain-PAD scores were compared between patients with OCD and HCs in dataset 1 using a two-tailed two-sample *t-*test. We also investigated whether there was a sex-related difference in the brain-PAD scores of patients with OCD by comparing the brain-PAD scores of female patients with those of male patients. Sex (not in sex difference), age, age2 (maybe also include TIV) and educational level were used as covariates in statistical models (19).

### Association With Clinical Symptoms

To explore the association between abnormal brain development and clinical symptoms in patients with OCD, Pearson's correlation coefficient was calculated for brain-PAD scores and symptoms, including duration of the disease and Y-BOCS scores.

### Validation

To exclude the effect of atlas selection on our results, we validated all results (unless otherwise stated) with an atlas of 200 regions of interest (37).

## Results

### Demographic Information

The demographic information for the dataset used in this study is included in Table 1. There was no significant difference between patients with OCD and HCs in terms of sociodemographic characteristics, such as age and sex. While, patients exhibited lower education level than HCs.

**Table 1:** Demographic and clinical characteristics of participants.

	**Dataset 1**		**P**	**Dataset 2**
	**HC (*N* = 106)**	**OCD (*N* = 90)**		**Subjects (*N* = 270)**
Male, No. (%)	53 (50.00)	50 (55.56)	0.440^a^	98 (36.30)
Age, mean (SD) [range], y	23.09 (5.63) [16–43]	22.58 (9.10) [12–49]	0.628^b^	31.50 (9.99) [19–50]
Educational level, mean (SD), y	15.18 (3.19)	11.81 (3.03)	<0.001^b^	-
Duration of illness, mean (SD), m	-	48.08 (57.61)		-
Y-BOCS score, mean (SD), [range]	-	21.92 (7.09) [1–40]		-

*Y-BOCS, Yale–Brown Obsessive Compulsive Scale*;

a *Chi-square t-test*;

b *two-tailed two sample t-test*;

*HC, healthy control; OCD, obsessive-compulsive disorder*.

### Performance of the Prediction Model

**Figure 1:**
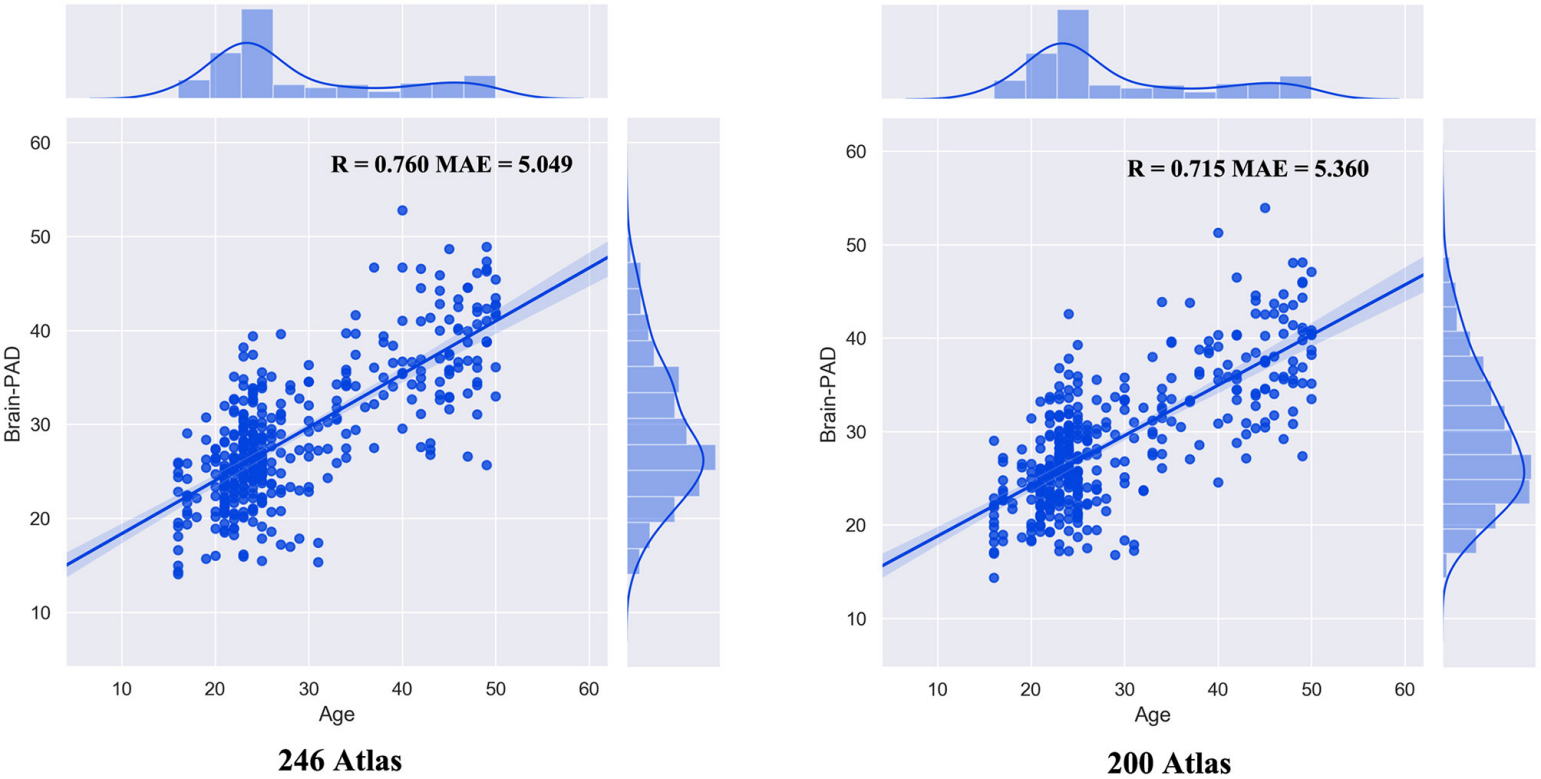
The performance of the prediction model.

**Figure 2:**
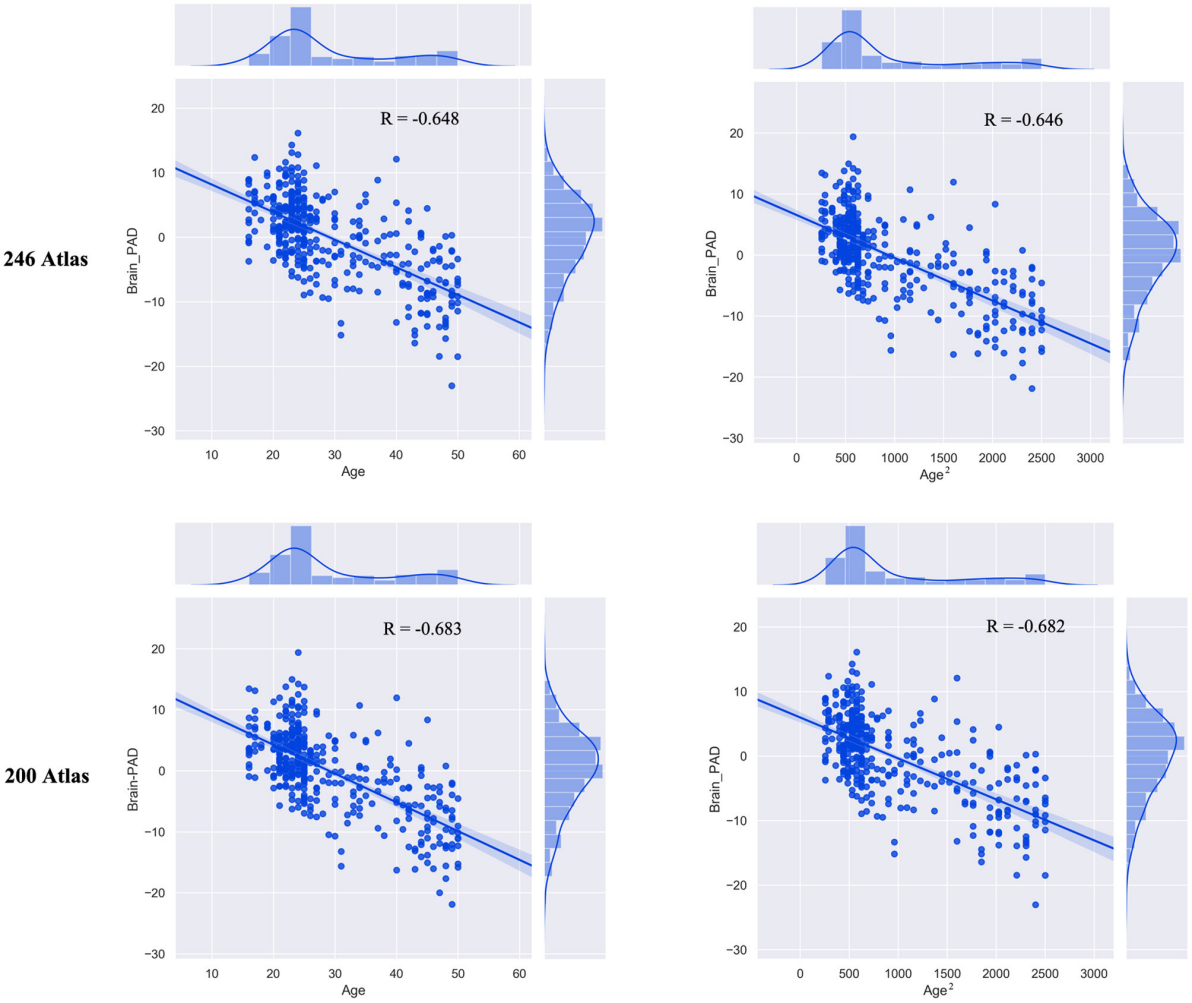
The correlation between brain-PAD scores and age (age^2^).

The built prediction model performed consistently with different atlas selections (Figure 1). The correlation between the chronological age and estimated brain age was 0.760 (MAE = 5.049) for the 246 atlas (R = 0.715; MAE = 5.360 for 200 atlas). Then, we observed significant correlation between brain-PAD scores and age (*r* = −0.655, *p* <0.001) and age^2^(*r* = −0.653, *p* <0.001). There was no significant correlation between TIV and brain-PAD score (*r* = −0.066, *p* = 0.200) (Figure 2).

### Accelerated Brain Age in Patients With OCD

**Figure 3:**
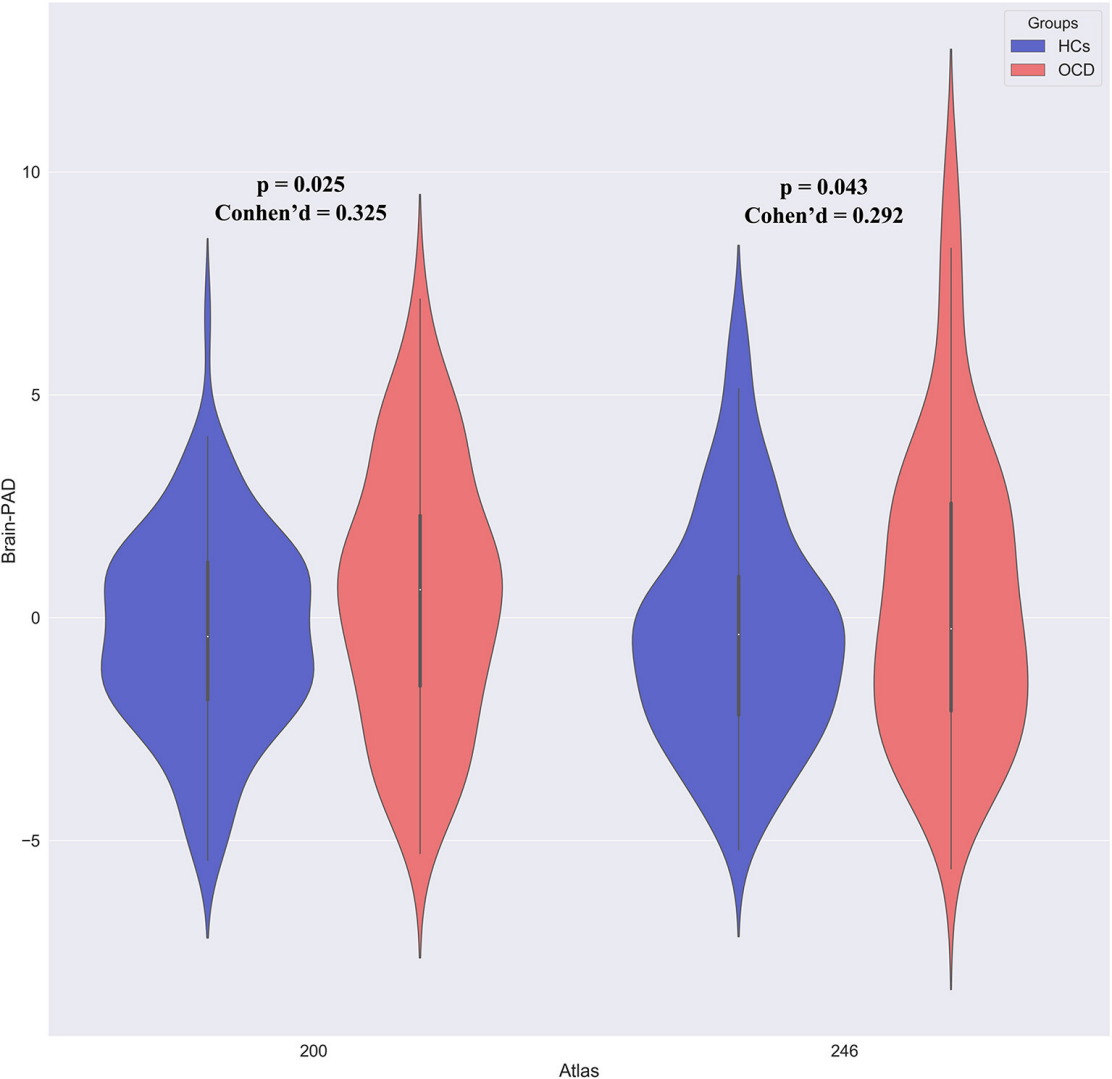
Aberrance of brain-PAD scores in patients with OCD.

To explore whether the brain development trajectory was abnormal in patients with OCD, we compared brain-PAD scores between patients with OCD and HCs. According to results above, we included age, age^2^ and educational level as covariates in this procedure. Patients with OCD had higher brain-PAD scores than HCs (+ 0.826 years, *t* = 2.266, *p* = 0.025, Cohen's d = 0.325), suggestive of accelerated maturation of the brain in patients with OCD (Figure 3). To eliminate the effect of atlas selection, we validated this result by using a different atlas (+ 0.847 years, *t* = 2.037, *p* = 0.043, Cohen's d = 0.292 for 200 atlas). In addition, brain-PAD score did not differ between male and female patients with OCD.

We further divided patients into two subgroups (subgroup 1 with age <=25 and subgroup 2 with age > 25) considering that the human brains reached a full maturation at the age of 25 (39). The positive brain-PAD scores in subgroup 1 suggested accelerated brain maturation while that in subgroup 2 suggested accelerated brain ageing. As a result, subgroup 1 exhibited significant larger brain-PAD scores than HCs (*t* = 2.800, *p* = 0.006, Cohen's d = 0.4411). However, there was no significant difference in terms of brain-PAD scores between subgroup 2 and HCs (*t* = 0.346, *p* = 0.730, Cohen's d = 0.077).

### Association With Clinical Symptoms

**Figure 4:**
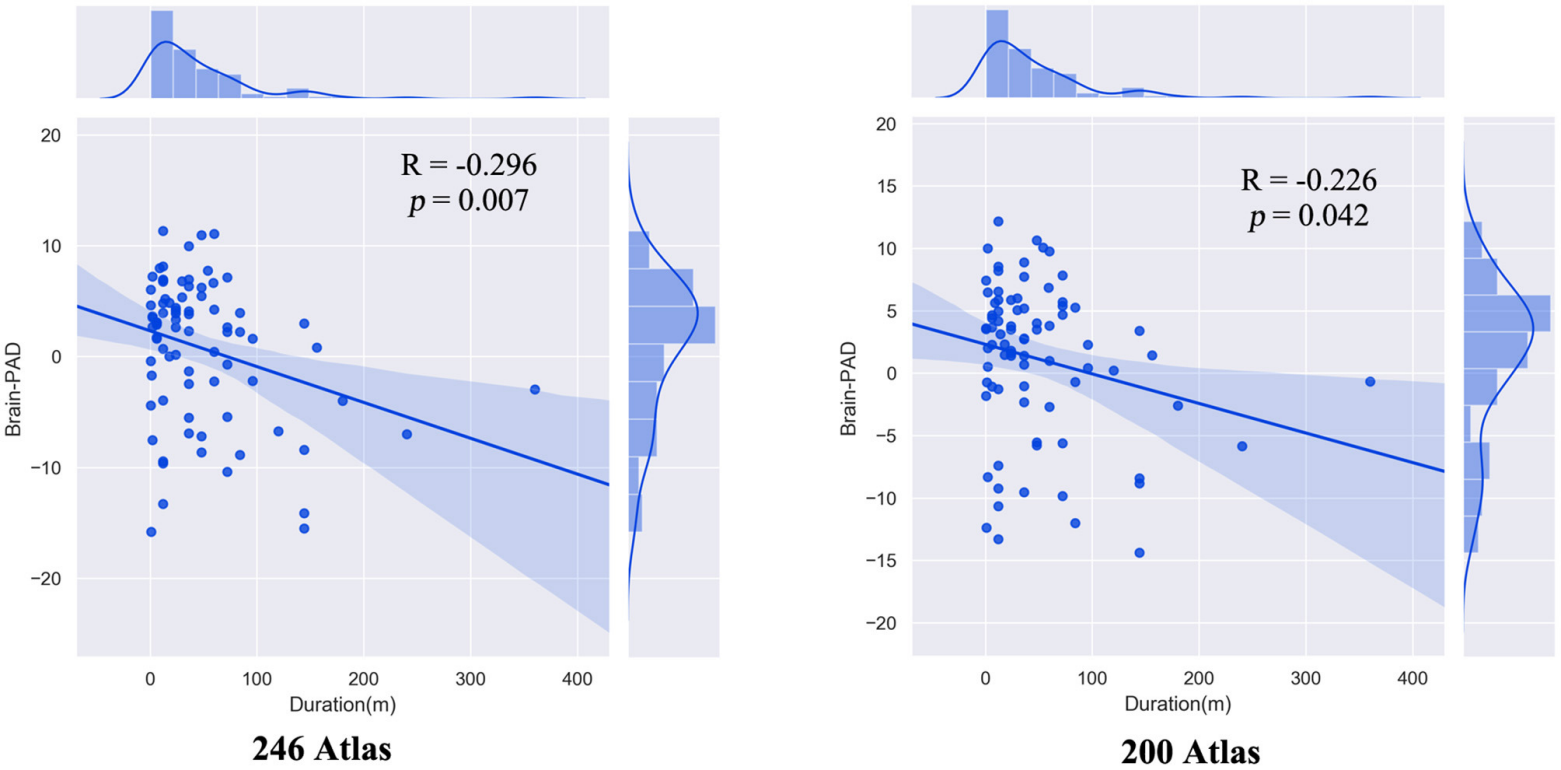
The correlation between brain-PAD scores and illness duration (month) in patients with OCD.

There was a significant negative correlation between brain-PAD scores and the duration of illness in patients with OCD. This result was validated using a different brain atlas (Figure 4). We did not observe significant correlation between Y-BOCS scores and brain-PAD scores (*r* = 0.045, *p* = 0.673)

## Discussion

In this study, we investigated whether brain development trajectory was abnormal in patients with OCD by adopting a brain age prediction model where the gray matter volumes of brain regions were used as features for the first time. We found that patients with OCD presented with higher brain-PAD scores than matched HCs, which suggests that there is accelerated maturation of the brain in these patients. In addition, the brain-PAD scores were negatively correlated with the duration of illness in patients with OCD but not with Y-BOCS scores. Combined with the finding that there was no significant difference between the sexes, these results suggest that accelerated maturation of the brain occurs at the beginning of the disease process and might be a trait-related aberrance of OCD. Overall, we observed an accelerated brain development in patients with OCD, a finding that could help us better understand the pathogenesis of OCD.

As we hypothesized, the brain-PAD scores in patients with OCD were higher than those in HCs (+ 0.826 years, *t* = 2.266, *p* = 0.025, Cohen's d = 0.325 for the 246 atlas), suggestive of accelerated predicted brain aging in patients with OCD. Major psychiatric diseases have been found to be associated with accelerated brain aging, including schizophrenia, depression, borderline personality disorder, and bipolar disorder (18–20). OCD was found to be highly comorbid with diseases such as schizophrenia, bipolar disorder, and depression (4, 5, 7–10, 40) suggestive of overlap in the pathogenesis of OCD with the pathogenesis of these diseases. Mental disorders are accompanied by accelerated aging trajectories, as supported by a large body of evidence (11–17) and confirmed by recent studies using the brain age method (18–20). However, whether OCD presents with a similar abnormal brain development trajectory had yet to be established. Our results show that OCD is associated with accelerated brain aging for the first time. In addition, although different mental disorders are associated with accelerated brain aging, the extent of acceleration has been shown to vary across different mental diseases [e.g., + 2.64 years for early-stage schizophrenia (18), + 1.08 years for depression (19)] and across stages of illness [e.g., + 1.7 years for participants at clinical risk for schizophrenia (20), + 3.36 (41) or 5.5 years for established illness (20)]. Considering the fact that the human brains reached a full maturation at the age of 25 (39), the meaning of positive brain-PAD scores was not different in older patients with age larger than 25. We further divided patients into subgroups according to age (subgroup1 with age <=25 and subgroup 2 with age >25). Subgroup 1 exhibited larger brain-PAD scores than HCs while not subgroup 2. Overall, the finding that OCD is associated with abnormal brain development deepens our understanding of the pathogenesis of OCD.

The duration of illness was found to be negatively correlated with brain-PAD scores in patients with OCD, which suggests that brain-PAD scores might capture progressive structural brain changes. Mental disorders, such as schizophrenia (42, 43), generalized anxiety disorder (44) and depression, (45) were found to be neuroprogressive illnesses accompanied by progressive structural brain changes throughout the disease course. The negative correlation hinted that higher brain age was a stage-dependent phenomenon in OCD. This phenomenon could mirror the transition from a clinically unstable period, with large variability in functioning, to a relatively stable period, when patients have reached a plateau in functioning (46, 47). The same phenomenon was also found in depression (27). Another possible explanation was the compensation mechanism considering that increased gray matter volume was also reported in OCD (27, 48). However, this correlation should be considered carefully, as there was limited number of patients with illness duration larger than 100 months. Further studies could test this hypothesis. In addition, the fact that there was no significant correlation between brain-PAD scores and Y-BOCS scores, as well as no significant difference between female and male patients, hinted that accelerated brain aging was independent of the severity of symptoms in OCD.

### Limitations and Future Directions

The findings of this study should be considered in light of some limitations. First, nicotine and alcohol use were not controlled for in the current study and they might also affect the brain-PAD scores (49). Future studies should take into account these factors. Second, our results were based on a single dataset; an external validation dataset was needed to confirm these results. Third, the negative correlation between duration of illness and brain-PAD scores suggested that the accelerated brain age might be more pronounced since the beginning of illness, and future studies could investigate this hypothesis. Forth, the educational level of patients and controls was not matched for two groups, and future studies could examine the association between education level and brain age. Fifth, as the first attempt to investigate abnormal brain age in OCD, we only used gray matter volume as features for its interpretability when constructing prediction model. However, future studies should take into account additional features including white matter volume, structural/functional connectome. Finally, adopting deep learning method would boost the performance of the prediction model (25, 50), future studies should use large dataset combined deep learning method if possible.

## Conclusion

To our knowledge, this is the first study to explore abnormal brain age in patients with OCD. We found that patients with OCD had higher brain-PAD scores than HCs, suggestive of accelerated brain development in patients with OCD. Moreover, an atypical brain development trajectory might be related to the progression of OCD and is independent of the severity of symptoms. These results suggest for the first time that there is accelerated brain aging in patients with OCD.

## Data Availability Statement

The raw data supporting the conclusions of this article will be made available by the authors, without undue reservation.

## Ethics Statement

The studies involving human participants were reviewed and approved by the Ethics Committee of the First Affiliated Hospital of Zhengzhou University. The patients/participants provided their written informed consent to participate in this study.

## Author Contributions

LL, SH, and JC designed the study. LY, BW, and XZ analyzed the data. LL, JC, and YZ drafted the work. All authors contributed to the article and approved the submitted version.

## Funding

This research study was supported by the Natural Science Foundation of China (81601467, 81871327, and 62106229), Medical science and technology research project of Henan province (201701011, SBGJ202102103, and SBGJ202101013) and The First Affiliated Hospital of Zhengzhou University (Grant No: YNQN2017160).

## Conflict of Interest

The authors declare that the research was conducted in the absence of any commercial or financial relationships that could be construed as a potential conflict of interest.

## Publisher's Note

All claims expressed in this article are solely those of the authors and do not necessarily represent those of their affiliated organizations, or those of the publisher, the editors and the reviewers. Any product that may be evaluated in this article, or claim that may be made by its manufacturer, is not guaranteed or endorsed by the publisher.
